# Subtalar Arthroereisis for Flexible Flatfoot in Children—Clinical, Radiographic and Pedobarographic Outcome Comparing Three Different Methods

**DOI:** 10.3390/children8050359

**Published:** 2021-04-30

**Authors:** Bjoern Vogt, Gregor Toporowski, Georg Gosheger, Jan Duedal Rölfing, Dieter Rosenbaum, Frank Schiedel, Andrea Laufer, Marie-Theres Kleine-Koenig, Christoph Theil, Robert Roedl, Adrien Frommer

**Affiliations:** 1 Pediatric Orthopedics, Deformity Reconstruction and Foot Surgery, University Hospital Muenster, 48149 Muenster, Germany; gregor.toporowski@ukmuenster.de (G.T.); andrea.laufer@ukmuenster.de (A.L.); robert.roedl@ukmuenster.de (R.R.); adrien.frommer@ukmuenster.de (A.F.); 2 General Orthopedics and Tumor Orthopedics, University Hospital Muenster, 48149 Muenster, Germany; georg.gosheger@ukmuenster.de (G.G.); christoph.theil@ukmuenster.de (C.T.); 3 Orthopaedic Reconstruction, Aarhus University Hospital, 8000 Aarhus, Denmark; jan.roelfing@rm.dk; 4 Institute of Experimental Musculoskeletal Medicine, Movement Analysis Laboratory, University Hospital Muenster, 48149 Muenster, Germany; diro@uni-muenster.de; 5 Pediatric Orthopedics and Neuroorthopedics, Clemenshospital Muenster, 48153 Muenster, Germany; f.schiedel@alexianer.de (F.S.); m.kleine-koenig@alexianer.de (M.-T.K.-K.)

**Keywords:** children, flexible flatfoot, subtalar arthroereisis, sinus tarsi implant, calcaneus stop screw, Kalix^®^ implant, Giannini implant, SESA, pedobarography

## Abstract

Subtalar arthroereises (STA) is a minimally invasive and reversible surgery to correct symptomatic flexible flatfoot (FFF) in children. Various techniques were described either applying expandable sinus tarsi implants or lateral calcaneus stop screws. Studies comparing the outcome of STA with different devices are rare. This retrospective single-center cohort study analyzes the results of STA using three different implants. 113 STA were performed in 73 consecutive patients (28 females). Mean age at surgery was 10.8 years (range 5–16). Mean follow-up was 29.0 months (range 1–111). In 21 feet the non-absorbable Kalix^®^ endorthesis and in 56 feet the absorbable Giannini endorthesis were applied. Subtalar extraarticular screw arthroereises (SESA) was conducted in 36 feet. Clinical, radiographic and pedobarographic parameters were analyzed. No intraoperative complications were observed. All three procedures achieved comparable improvements of the clinical, radiographic and pedobarographic parameters. The mean foot function index (FFI) improved from 36.4 (range 12–63) to 22.8 (range 2–55). The mean preoperative calcaneal inclination angle and the lateral talocalcaneal angle improved from 9.5° (range 0–22) and 42.3° (range 21–62) to 12.8° (range 0–26) and 37.6° (range 15–56), respectively. Pedobarographically determined values of the arch index, the medial midfoot contact area and the medial forefoot peak pressure decreased. In contrast to SESA (1/36, 3%), a higher incidence of implant-related complications was observed using Kalix^®^ (6/21, 29%) and Giannini (10/56, 8%) sinus tarsi implants. Peroneal muscle contractures only occurred in the SESA group (4/36, 11%). Premature removal due to treatment-related complications was necessary in 6/21 Kalix^®^ implants (29%), 4/56 Giannini implants (7%) and 4/36 SESA implants (11%). Implant choice for treatment of painful FFF in children with STA seems to play a subordinate role. Clinical, radiographic and pedobarographic outcomes are comparable between the applied implants. Surgeons and patients should be aware of the different spectrum of implant-related complications. Treatment can be reliably monitored by radiation-free pedobarography providing dynamic information about the deformity.

## 1. Introduction

Flexible flatfoot (FFF) in children and adults is a common reason for orthopedic consultation [1]. To date, the distinction between physiological and pathological FFF remains challenging, especially in growing patients [2]. Standard assessment includes clinical and radiological examination. Modern technology such as digital pedobarography has helped to improve understanding and treatment of the deformity [3,4,5].

When FFF becomes symptomatic, affecting gait and limiting quality of life, different treatment options are available. If conservative treatment such as weight reduction, physiotherapy or insoles fail, operative treatment can be considered [2,6,7,8]. Surgical approaches include soft-tissue procedures and bony corrections such as subtalar arthroereises (STA), correction osteotomies and arthrodeses [7,9,10,11,12,13].

STA is a minimally invasive surgical option and has previously shown good results for correction of pediatric FFF [6,13,14]. In contrast to arthrodeses or correction osteotomies, STA is reversible maintaining motion of the subtalar joint during and after treatment. For STA self-locking absorbable [8,14,15] or non-absorbable [16,17,18,19] devices implanted in the sinus tarsi acting as a plug as well as impact blocking subtalar extraarticular screw arthroereises (SESA) [6,12,20] can be applied. While pedobarography is an established method for the analysis of foot deformity, its role in assessing the outcome of STA performed in patients with FFF remains to be further elucidated [4,5].

The aim of this study was to compare the outcome of surgical FFF treatment with three different types of STA implants comparing clinical, radiographic and pedobarographic parameters.

## 2. Materials and Methods

### 2.1. Patients and Indications

A single center, retrospective cohort study was conducted including all patients consecutively treated with STA for FFF from 2002 until 2013. 113 STA were performed in 73 patients (28 females, 38%) to correct idiopathic (*n* = 48, 65.8%) or neuromuscular (*n* = 25, 34.2%) FFF. The mean age at surgery was 10.8 years (range 5–16). The mean follow-up was 29.0 months (range 1–111). The average body mass index (BMI) at surgery was 20.5 kg/m^2^ (range 15.4–25.0) (Table 1).

During the first consultation for painful FFF, patients were advised to intensify conservative treatment for a minimum of three months and were only considered for STA if conservative treatment failed to relief symptoms. All patients with neuromuscular disease had a preoperative ambulation level of I or II according to the Gross Motor Function Classification System (GMFCS) [21]. STA was exclusively performed if active correction of the foot was possible and clinical, radiographic and pedobarographic evaluation confirmed FFF pattern. STA was not conducted in patients older than 16 years, with a BMI > 25, with rigid flat foot deformity, tarsal coalition or a GMFCS level > 2.

### 2.2. Implants

**Figure 1:**
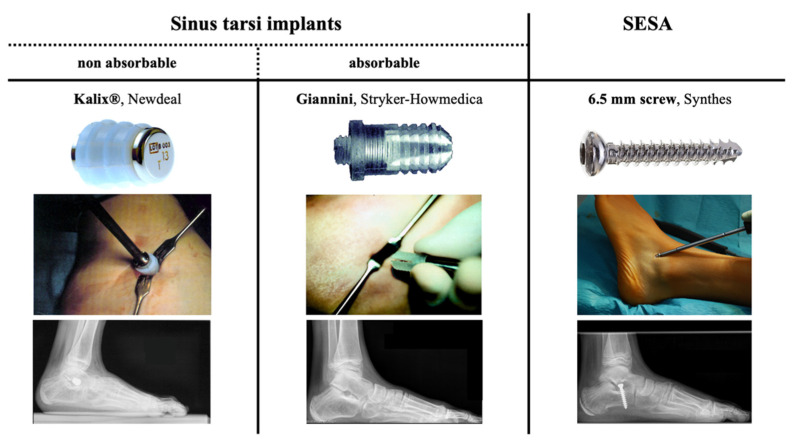
Used subtalar arthroereises implants throughout the study showing the devices, the intraoperative applications and the postoperative lateral radiographs. Sinus tarsi implants: Non-absorbable Kalix^®^ endorthesis (**left**), absorbable Giannini endorthesis (**center**). Subtalar extra-articular screw arthroereises (SESA) with 6.5 mm cancellous screw (**right**). The pictures of the devices were provided courtesy of the companies.

In the studied cohort STA was consecutively performed with three different types of implants. From 2000–2002 the non-absorbable Kalix^®^ sinus tarsi endorthesis (Newdeal, Lyon, France) was used in 21 feet. The Kalix^®^ implant was abandoned when the absorbable Giannini sinus tarsi endorthesis (Stryker-Howmedica, Kalamazoo, MI, USA) was available. From 2002–2010 the Giannini implant was applied in 56 feet. In 2010 the device was temporary taken off the market due to observed adverse effects such as dislocation and breakage leading to a change of paradigm in our department. Since 2010 exclusively SESA was conducted using a 6.5 mm non-canulated cancellous screw (Synthes, West Chester, PA, USA) as proposed by De Pellegrin [6,12]. 36 feet treated with SESA until 2013 were included in this study (Figure 1).

### 2.3. Pre- and Postoperative Assessment

All patients underwent clinical, radiographic and pedobarographic examination preoperatively and at the end of treatment. In patients treated with SESA and the Kalix^®^ endorthesis postoperative radiographic and pedobarographic measurements were conducted three months after implant removal (=end of treatment). In patients treated with the Giannini endorthesis the postoperative measurements were made two years after surgery corresponding to the removal time of SESA and the Kalix^®^ endorthesis.

Patients were asked to evaluate their overall treatment satisfaction using absolute category rating with the levels excellent, good, fair and poor. Patient reported outcome was measured using the foot function index (FFI) which in total comprises 23 items (FFI-T) separated into a pain (FFI-P), disability (FFI-D) and activity limitation (FFI-A) subscale [22].

**Figure 2:**
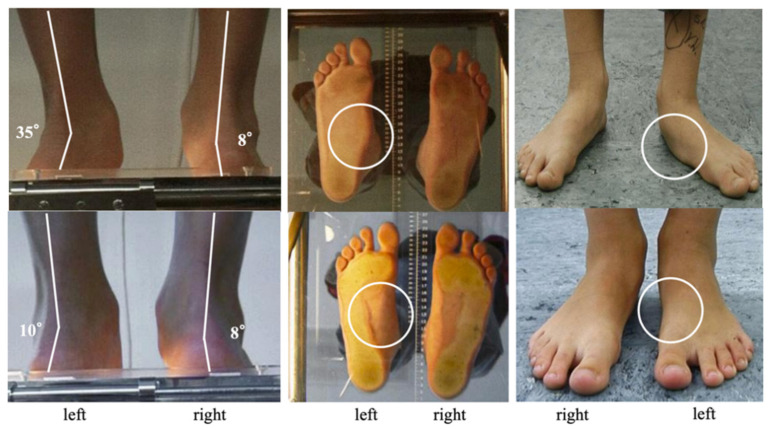
Pre- and postoperative clinical and podoscopic assessment. A 12-year-old boy received a subtalar extra-articular screw arthroereises (SESA) for painful unilateral flexible flatfoot (FFF) on the left side. While preoperatively typical FFF pattern was noticeable (**top**) the postoperative clinical and podoscopic examination depicted a good correction of the hindfoot valgus and the medial arch (**bottom**) (same patient as shown in Figure 3, Figure 4 and Figure 5).

**Figure 3:**
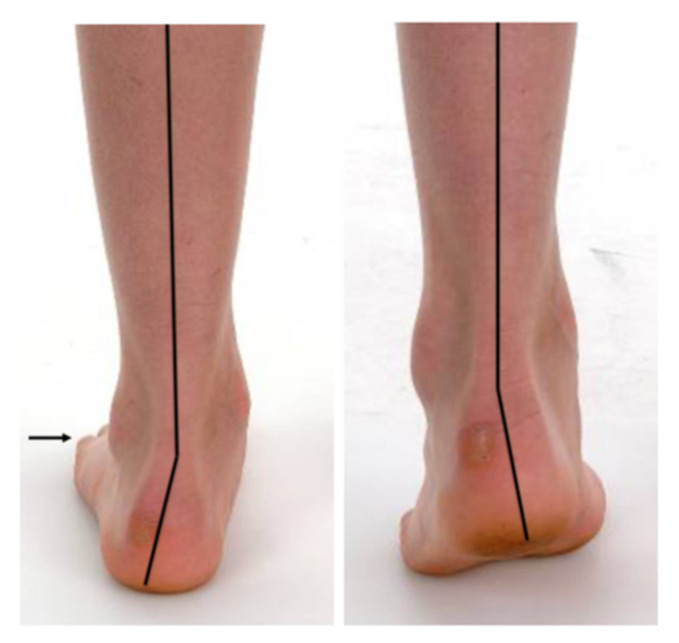
Functional assessment of the flexibility of the foot deformity. Hindfoot valgus and forefoot abduction (➔ positive “too many toes-sign”) (**left**). Complete active correction of the hindfoot valgus showing restored heel inversion (**right**) (same patient as shown in Figure 2, Figure 4 and Figure 5).

Hindfoot alignment in relation to the lower leg, the medial foot arch and the “too many toes-sign” were assessed clinically on a podometer. Preoperative active correction of the foot deformity was determined using the “heel rise test” (Figure 2 and Figure 3).

**Figure 4:**
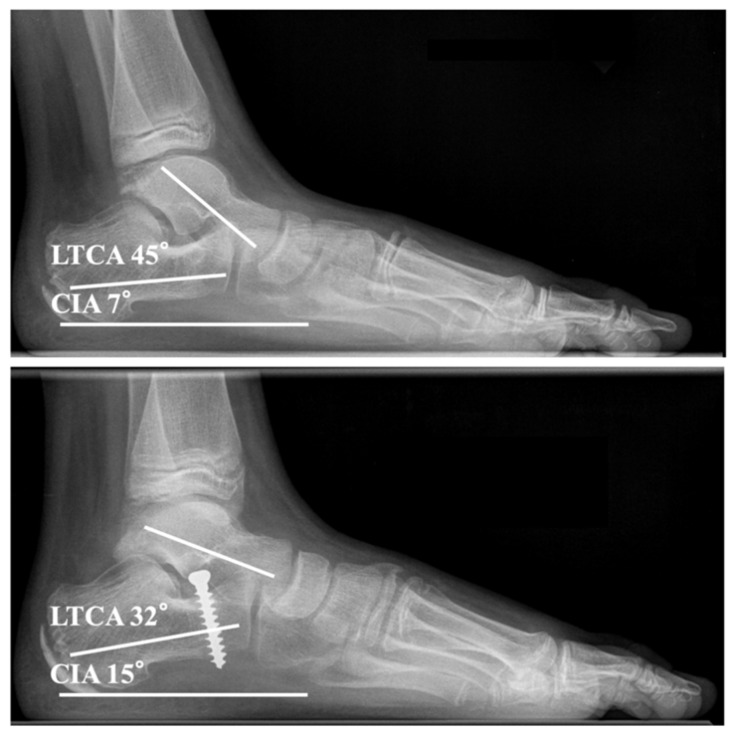
Full weight bearing lateral radiographs before and after subtalar extra-articular screw arthroereises: Improvement of the calcaneal inclination angle (CIA) and lateral talocalcaneal angle (LTCA) from 7° and 45° before surgery (**top**) to 15° and 32° after surgery (**bottom**), respectively (same patient as shown in Figure 2, Figure 3 and Figure 5).

Anteroposterior and lateral full weight bearing radiographs of the feet were obtained. All measurements were conducted on calibrated images with the PACS^®^ system (GE-Healthcare, Chicago, IL, USA). Selected radiographic parameters such as the calcaneal inclination angle (CIA) and the lateral talocalcaneal angle (LTCA) were measured preoperatively and at follow-up (Figure 4).

**Figure 5:**
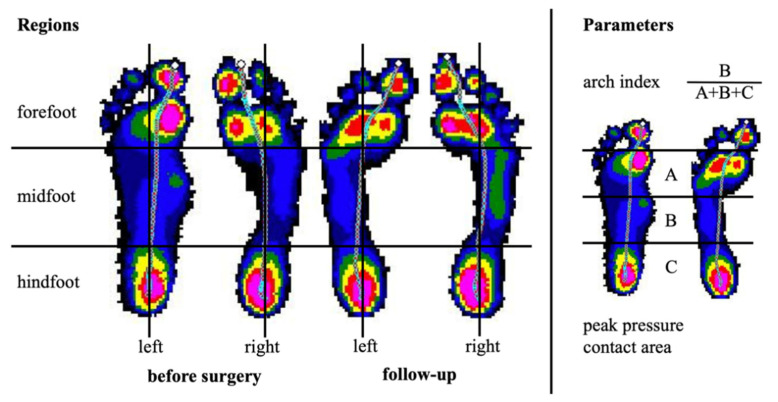
Pedobarographic examination before and after subtalar arthroereises (STA): The foot was longitudinally divided into a medial and lateral zone and transversally divided into a forefoot, midfoot and hindfoot zone resulting in six regions of interest (**left**). Considering flexible flatfoot patterns selected parameters and regions were evaluated (arch index, medial midfoot contact area and medial forefoot peak pressure) (**right**). The pedobarographic analysis demonstrates the reconstitution of physiological and symmetrical foot alignment after STA on the left side (same patient as shown in Figure 2, Figure 3 and Figure 4).

For pedobarographic assessment of dynamic foot loading patients walked barefoot over the EMED^®^ ST4 pressure distribution platform (Novel GmbH, Munich, Germany; 4 sensors/cm^2^, 50 Hz). Measurements were conducted during mid-gait at a self-selected walking speed. Five steps of the left and five steps of the right foot were recorded. For data analysis the database Medical Professional (Version 12.2.7, Novel GmbH, Munich, Germany) was used. Selected pedobarographic parameters such as the arch index [23], the medial midfoot contact area and the medial forefoot peak pressure [4,5] were assessed preoperatively and at follow-up (Figure 5).

### 2.4. Surgical Technique, Perioperative Management and Follow-Up

All surgeries were either conducted or supervised by B. V., F. S. or R. R. Bilateral operations were conducted simultaneously. Patients were placed in a supine position with a tourniquet on thigh level.

Ankle range of motion was assessed by the “Silfverskiöld test” with the patient under general anesthesia [24]. If passive ankle dorsiflexion was limited to 0° with flexed hip and knee (51/113 operated feet, 45%) either an aponeurotic gastrocnemius recession (Strayer technique [25]) or a percutaneous lengthening of the Achilles tendon (Hoke technique [26]) was concomitantly performed according to the surgeon’s preference prior to STA.

A minimally invasive incision was placed 0.5–1 cm distal to the lateral malleolus over the sinus tarsi. The Kalix^®^ and Giannini sinus tarsi implants were inserted as previously described (Figure 1) [15,16]. For SESA, the foot was held in maximum plantarflexion and supination for implant insertion. According to De Pellegrin a 6.5 mm non-cannulated cancellous screw was inserted into the lateral calcaneus (Figure 1) [6]. Implant positioning was controlled intraoperatively by image intensifier and correction was clinically assessed with the “plantar malleoli view sign” [6].

A synthetic cast was applied for two weeks postoperatively and full weight bearing was permitted immediately after surgery. Patients were followed in the outpatient clinic periodically every six months. Removal of non-absorbable implants was routinely scheduled after a minimum treatment period of two years. Thus, 15/21 (71%) Kalix^®^ and 32/36 (89%) SESA implants were explanted after a mean time of 28.8 months (range 18–111). Preliminary implant removal was necessary in 14/113 (12%) feet.

### 2.5. Statistical Report

Descriptive statistics were performed using means and range (minimum/maximum) for continuous variables. Pre- and postoperative data were analyzed using the Wilcoxon signed-rank test, the Mann-Whitney U test and the Kruskal-Wallis test. Contingency analysis was performed by 2 × 2 and 2 × 3 Chi-squared tests. The level of significance was set at α < 0.05. The statistical tests were performed using Prism, v7.00 (GraphPad, San Diego, CA, USA).

## 3. Results

### 3.1. Clinical Outcome

Treatment satisfaction was comparable between the devices and scored excellent in 14/73 (19%) patients, good in 39/73 (53%) patients, fair in 13/73 (18%) patients and poor in 7/73 (10%) patients (Table 2).

Complete pain relief was documented in 102/113 (90%) feet, while persistent pain was reported in 1/21 (5%) feet with the Kalix^®^ implant, in 5/56 (9%) feet with the Giannini implant and in 5/36 (14%) feet with SESA at latest follow-up (*p* = 0.512).

Clinical signs of under-correction or recurrent deformity were observed in the 17 (15%) feet with implant-related complications, whereas 96/113 (85%) feet showed no signs of under-correction in terms of persistent hindfoot valgus or over-correction with hindfoot varus after surgery.

**Figure 6:**
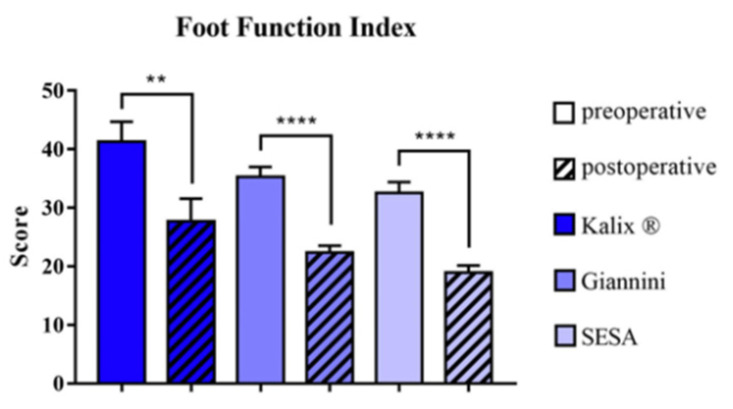
Pre- and postoperative total foot function index. Patient treatment satisfaction was measured before and at the end of treatment with Kalix ^®^ implants, Giannini implants or subtalar extraarticular screw arthroereises (SESA). (μ ± SEM, Wilcoxon signed-rank test, ** = *p* < 0.01, **** = *p* < 0.0001).

The mean FFI-T, FFI-P, FFI-D and FFI-A improved statistically significantly from 36.4 (range 12–63), 40.9 (range 14–72), 42.6 (range 5–78) and 11.6 (range 2–26) before surgery to 22.8 (range 2–55), 26.8 (range 0–66), 26.2 (range 0–69) and 6.2 (range 0–23) at follow-up, respectively. No implant-dependent statistically significant differences concerning FFI-T (*p* = 0.992), FFI-P (*p* = 0.953), FFI-D (*p* = 0.999) or FFI-A (*p* = 0.985) were observed (Figure 6).

### 3.2. Radiographic Outcome

**Figure 7:**
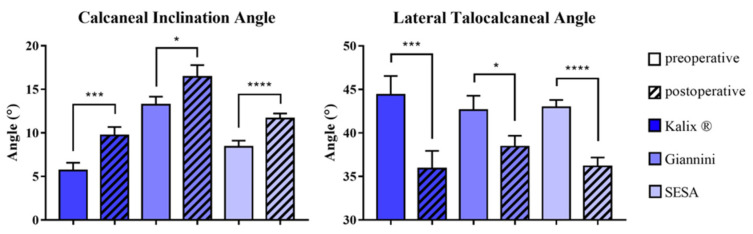
Comparison of pre- and postoperative radiographic foot alignment parameters subclassified by implant type. Calcaneal inclination angle (CIA) (**left**), Lateral talocalcaneal angle (LTCA) (**right**). (μ ± SEM, Wilcoxon signed-rank test, * = *p* < 0.05, *** = *p* < 0.001, **** = *p* < 0.0001). SESA: subtalar extraarticular screw arthroereises.

The mean CIA and LTCA improved statistically significantly from 9.5° (range 0–22°) and 42.3° (range 21–62°) before surgery to 12.8° (range 0–26°, *p* < 0.001) and 37.6° (range 15–56°, *p* < 0.001) at follow-up, respectively. Irrespective of the implant the CIA and LTCA showed statistically significant improvements. No implant-dependent statistically significant differences concerning correction of CIA (*p* = 0.631) and LTCA (*p* = 0.437) were observed (Figure 7).

### 3.3. Pedobarographic Outcome

**Figure 8:**
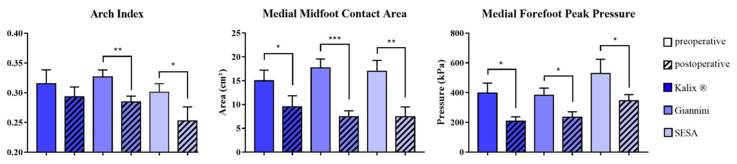
Pre- and postoperative pedobarographic measurements depending on the used implant type. Arch index (**left**), medial midfoot contact area (**center**) and medial forefoot peak pressure (**right**) are shown. (μ ± SEM, Wilcoxon signed-rank test, * = *p* < 0.05, ** = *p* < 0.01, *** = *p* < 0.001). SESA: subtalar extraarticular screw arthroereises.

The mean arch index, contact area medial midfoot and peak pressure medial forefoot statistically significantly improved from 0.32 (range 0.06–0.44), 16.7 cm^2^ (range 0.3–34.4) and 417.3 kPa (range 18–1204) before surgery to 0.28 (range 0.06–0.41, *p* < 0.001), 8.0 cm^2^ (range 0.1–35.0, *p* < 0.001) and 244.5 kPa (range 31–552, *p* < 0.001) at follow-up, respectively. Postoperative pedobarographic measurements showed a statistically significant improvement within each group except for the arch index in the Kalix^®^ group. No implant-dependent statistically significant differences concerning correction of the arch index (*p* = 0.346), midfoot contact area (*p* = 0.542) and medial forefoot peak pressure (*p* = 0.874) were observed (Figure 8).

### 3.4. Complications

No intra- and postoperative complications concerning neurovascular damage, delayed wound healing, infection or fracture occurred. Implant-related complications such as primary malposition and secondary dislocation or breakage of the implant were recorded in 17/113 (15%) feet. Compared to SESA a significantly higher incidence of implant-related complications was observed using Kalix^®^ (*p* = 0.004) and Giannini implants (*p* = 0.029). The reasons for treatment-related pain were different between the devices. While painful peroneal muscle contractures were only observed after SESA in 4/36 feet (11%), pain due to implant-related complications was numerically more frequent using the Kalix^®^ (*p* = 0.013) and Giannini endortheses (*p* = 0.101) (Table 3).

**Figure 9:**
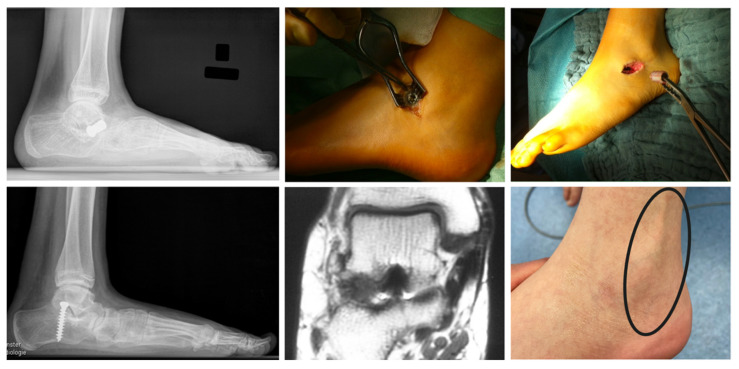
Treatment-related complications. Secondary dislocation of a Kalix^®^ endorthesis depicted on lateral radiograph showing the dislocated implant and recurrent deformity (**top**–**left**) and intraoperative situation during premature removal surgery (**top**–**center**, **right**). Primary malposition of a subtalar extra-articular screw arthroereises (SESA) depicted on lateral radiograph showing the wrong screw position and recurrent deformity (**bottom**–**left**). Secondary breakage of a Giannini implant depicted on a coronar magnetic resonance imaging scan (**bottom**–**center**). Clinical presentation of a reactive peroneal muscle contracture after SESA (**bottom**–**right**).

In the entire study cohort premature implant removal was necessary in 14/113 (12%) feet. 6/21 (29%) Kalix^®^ implants and 4/56 (7%) Giannini endortheses were prematurely removed due to implant-related complications. 3/17 (18%) observed implant-related complications were clinically inconsequential and did not require premature implant removal. In all 4/36 (11%) patients with peroneal muscle contracture after SESA the screws were prematurely explanted (Table 3, Figure 9).

## 4. Discussion

After failed conservative treatment of FFF in children, it remains challenging for surgeons and patients to agree on the preferrable surgical method. Due to its minimal invasiveness and promising results, STA has become an established procedure to treat FFF in growing patients, especially in European countries [2,6,14]. The operative techniques and the applied implants have constantly evolved since the first description of lateral arthroereises in 1970 [27]. The mechanical support by the implant as well as the improvement of proprioception during the treatment are thought to be the main mechanisms correcting the foot deformity by STA [2]. Independent of the type of implant, STA should only be performed if active correction of the foot deformity is possible [2,6].

To our knowledge, this is the first study comparing the clinical, radiographic and pedobarographic outcome after STA with all main types of available implants (absorbable and non-absorbable sinus tarsi implants and SESA). In the studied cohort we observed an improvement of clinical and radiographic parameters after treatment with STA irrespective of the applied implant. The findings are consistent with observations from other study groups describing an improvement of CIA and LTCA after STA with different types of implants [6,8,14,28,29]. Due to the variety of available devices a comparison between implants assessing advantages and disadvantages remains difficult. To date, only two studies have retrospectively compared the outcome after STA with different types of implants [13,30]. In accordance with our observations, both studies found comparable clinical and radiographic results concerning correction of the deformity irrespective of the chosen implant. Scialpi et al. assessed the results of STA performed with Giannini implants (21 patients, 40 feet) and SESA (22 patients, 40 feet) and described good results of treatment in 55–60% of cases but did not provide information about potential complications [13]. Baker et al. analyzed STA conducted with different types of absorbable (61 feet) and non-absorbable (32 feet) sinus tarsi implants and in contrast to our findings found similar rates of premature implant removal (17% and 19%) mainly due to implant-related pain [30].

Radiographic assessment is limited to a static and two-dimensional perception of deformity. This study provides new insights in diagnostical approaches by using pedobarography which helps to dynamically assess foot alignment. In addition to clinical examination pedobarography improves the three-dimensional understanding of FFF and its treatment [3,4,5]. Comparable studies to discuss the observation in the context of actual literature are rare. In fact, only two studies focused on pedobarographic changes after SESA [4,5]. In accordance with the results of these studies our findings demonstrate that functional and morphogenetic changes such as elevation of the longitudinal arch, decrease of medial midfoot contact and medial forefoot peak pressure after STA can reliably be assessed by pedobarography.

Due to the wide physiological range described for CIA and LTCA, pre- and postoperative radiographic measurement is insufficient to evaluate success of STA [31,32]. In our study cohort the mean LTCA lies within physiological ranges before surgery and at follow-up. In contrast to radiographic evaluation pedobarography bears the advantage to be radiation-free and to provide dynamic information of the foot deformity [4,5]. However, its availability can be limited depending on the hospital infrastructure. Nonetheless, one should not underestimate the established role of conventional radiographic examination since it helps to rule out structural flatfoot deformities such as tarsal coalitions and vertical talus.

Surgeons must be aware of the different spectrum of complications observed when performing STA with sinus tarsi implants or SESA. The rates of implant-related complications and premature implant removal in our cohorts are in accordance with previous studies that investigated the outcome of STA with the non-absorbable Kalix^®^ endorthesis [16,17,18,19], the absorbable Giannini implant [8,13,14,15,30] and SESA [6,12,13,20,31] and reported frequencies ranging from 1–32% [6,7,14,30,33]. On basis of these findings and the observations in this study implant-related complications leading to premature hardware removal occurred more frequently using sinus tarsi endortheses compared to SESA.

On the other hand, the development of peroneal muscle contractures appears to be a specific postoperative complication during treatment with SESA [30,34]. De Pellegrin et al. described peroneal muscle contractures due to an antalgic position in pronation in 14/485 patients treated by SESA (3%) and etiologically suspected a reaction to pain and to stimulation of the sinus tarsi mechanoreceptors, referring to the studies by Rein et al. [6,35,36]. In contrast to our own management consisting of premature implant removal in all affected patients, De Pellegrin et al. successfully resolved this complication in a conservative manner by physiotherapy, casts or orthoses and local injections [6]. Hamel observed this problem in 4/41 feet treated by SESA (10%) in 30 patients and assumed an inaccurate screw positioning as a possible cause of a dysfunctional phenomenon due to reactive peroneal muscle activation [34].

The precise positioning of the implant seems to be of crucial importance rather than the implant choice. While local soft tissue inflammation associated with implant absorption has been described, the application of absorbable implant spares an additional operation for hardware removal [14,33]. On the other hand, SESA does not compromise proprioceptive nerve endings and mechanoreceptor located in the sinus tarsi, which might be regarded as an advantage compared to sinus tarsi implants [35,36].

To date, there is no clear consensus about the preferrable surgical treatment for painful FFF [37,38]. While there is Consensus that corrective surgery is rarely necessary (presumably in less than 10% of patients), the perception of the surgical approach differs especially between North American surgeons and surgeons outside the US. Especially in European countries STA is more frequently applied [37,38]. The results from surgeon surveys conducted by the American Orthopaedic Foot and Ankle Society and European Paediatric Orthopaedic Society underline the need for international, prospective large cohort trials to identify the diagnostic and treatment leading to the best outcome.

Previous studies have analyzed if obesity influences the outcome of STA compared to normal weight [39,40]. However, no specific conclusion can be drawn based on the findings of this study since patients were only included with a BMI ≤ 25. One can presume that contrary to patients with normal weight, pedobarography is not subject to the same reliability when analyzing obese patients.

This study has several limitations due to its retrospective character and is biased by different cohort sizes, availability of the implants and uneven distribution of the etiologies. Patients were only included until 2013 since due to logistic reason pedobarographic analysis was inconsistently available after 2013. STA implants were used consecutively due to their availability and not during the same period. Time related parameters such as the surgeon’s expertise, evolution of implants and improved outcome measures with time might limit comparability of the studied cohorts. The statistical findings of this study should carefully be interpreted. We encourage readers to focus more on the clinical, radiographic and pedobarographic outcomes, rather than statistical comparisons and *p*-values. The fact that some patients were treated bilaterally and others unilaterally can limit comparability. Some statistical findings might not be applicable to every patient group. Most patients were immature at the time of implant removal or last follow-up and the study lacks long-term observation after treatment focusing on maintenance of the correction and occurrence of recurrent deformity. Furthermore, this study does not provide a control group to compare the outcome with the natural development of pediatric foot shape over time. Prospective randomized trials are needed to compare the benefits and disadvantages of the available implants with a long-term follow-up.

## 5. Conclusions

Independent of the type of implant, STA is a reliable surgical treatment for symptomatic FFF in children relieving pain and correcting deformity by dynamic and proprioceptive mechanisms. In contrast to two-dimensional and static radiographs, pedobarography provides additional dynamic information about the deformity and helps to monitor the outcome of STA.

Complications related to STA are rare. Painful peroneal muscle contracture seems to be a SESA-specific functional complication while implant-related complications occur more frequently during treatment with sinus tarsi implants. Surgeons should be aware of the different spectrum of complications when counseling patients about STA.

Since all three procedures achieved comparable improvements of the outcome measurements, implant choice for treatment of painful FFF in children with STA seems to play a subordinate role and should be left to the preference of the surgeon.

## Figures and Tables

**Table 1:** Study population.

	Total	Kalix^®^	Giannini	SESA
Number (patients/feet)	73/113	11/21	37/56	25/36
Age at operation in years (range)	10.8 (5–16)	10.2 (6–14)	10.3 (5–16)	12.0 (8–16)
Gender (male/female)	45/28	6/5	24/13	15/10
Etiology (idiopathic/ neuromuscular)	48/25	3/8	26/11	19/6
Side (right/left) (bilateral)	52/61 (40)	10/11 (10)	27/29 (19)	15/21 (11)
Body mass index in kg/m^2^ (range)	20.5 (15.4–25.0)	20.5 (16.3–25.0)	20.9 (18.5–24.2)	19.9 (15.4–24.8)
Lengthening of gastrocnemius muscle or Achilles tendon (yes/no)	51/62	14/7	22/34	15/21
Follow up in months (range)	29.0 (1–111)	29.6 (1–86)	30.1 (1–79)	27.0 (2–111)

Flexible flatfeet treated with non-absorbable Kalix^®^ implants, absorbable Giannini implants and subtalar extraarticular screw arthroereises (SESA).

**Table 2:** Treatment satisfaction.

	Total	Kalix^®^	Giannini	SESA	*p* Value
Excellent	14/73 (19.2%)	2/11 (18.2%)	7/37 (18.9%)	5/25 (20.0%)	0.990
Good	39/73 (53.4%)	6/11 (54.5%)	20/37 (54.1%)	13/25 (52.0%)	0.984
Fair	13/73 (17.8%)	2/11 (18.2%)	7/37 (18.9%)	4/25 (16.0%)	0.981
Poor	7/73 (9.6%)	1/11 (9.1%)	3/37 (8.1%)	3/25 (12.0%)	0.979

Comparing Kalix^®^ implants, Giannini implants and subtalar extraarticular screw arthroereises (SESA) (Chi-squared test).

**Table 3:** Complications.

	Total	Kalix^®^	Giannini	SESA
Implant-related complications	17/113 (15.0%)	6/21 (28.6%)	10/56 (17.9%)	1/36 (2.8%)
- Primary mal-position	1/113 (0.9%)	0/21 (0%)	0/56 (0%)	1/36 (2.8%)
- Secondary dislocation or breakage	16/113 (14.1%)	6/21 (28.6%)	10/56 (17.9%)	0/36 (0%)
Treatment-related pain based on	12/113 (10.6%)	4/21 (19.0%)	4/56 (7.1%)	4/36 (11.1%)
- Implant-related complications	8/113 (7.1%)	4/21 (19.0%)	4/56 (7.1%)	0/36 (0%)
- Peroneal muscle contractures	4/113 (3.5%)	0/21 (0%)	0/56 (0%)	4/36 (11.1%)
Premature removal	14/113 (12.4%)	6/21 (28.6%)	4/56 (7.1%)	4/36 (11.1%)

Comparing Kalix^®^ implants, Giannini implants and subtalar extraarticular screw arthroereises (SESA).

## Data Availability

The datasets generated during or analyzed during the current study are available from the corresponding author on reasonable request.
